# Integrating animal cruelty exposure into person-centered models of childhood adversity: latent classes and associations with depression, anxiety, and stress

**DOI:** 10.3389/fpsyt.2025.1701584

**Published:** 2025-11-07

**Authors:** Shelby E. McDonald, Camie Tomlinson, Nicole Nicotera, Lori R. Kogan, Tiarra Abell, Jada Ford, Sohaila Jafarian, Gehena Girish, Lydia Soto Rodriguez, Charlotte L. Bright

**Affiliations:** 1 School of Social Work, Colorado State University, Fort Collins, CO, United States; 2 Kent School of Social Work and Family Science, University of Louisville, Louisville, KY, United States; 3 Graduate School of Social Work, University of Denver, Denver, CO, United States; 4 Clinical Sciences, College of Veterinary Medicine, Colorado State University, Fort Collins, CO, United States; 5 Department of Psychological & Brain Sciences, University of Louisville, Louisville, KY, United States

**Keywords:** animal cruelty, adverse childhood experiences, interpersonal violence, mental health, latent class analysis, childhood adversity, violence prevention

## Abstract

**Background:**

Animal cruelty is a recognized correlate of interpersonal violence within family systems, yet children’s exposure to this form of harm remains absent from most standardized assessments of childhood adversity. Guided by the Dimensional Model of Adversity and Psychopathology, this study examined the co-occurrence of exposure to animal cruelty with other threat-related adverse childhood experiences (ACEs) and how these patterns relate to adult mental health.

**Methods:**

Our sample included 1,072 U.S. adults recruited online through Prolific, a licensed participant recruitment firm, using its representative U.S. sample option. Participants reported on childhood experiences of emotional abuse, physical abuse, sexual abuse, exposure to domestic violence, and exposure to animal cruelty, along with current symptoms of depression, anxiety, and stress. Latent class analysis identified subgroups based on adversity profiles. Associations between class membership and sociodemographic factors were examined, and differences in mental health outcomes across classes were estimated adjusting for these covariates.

**Results:**

A three-class model best fit the data: *low adversity* (29.6%), *interpersonal violence only* (34.8%), and *interpersonal violence and animal cruelty* (35.6%). Membership in the latter was more likely among younger participants, those with a minoritized gender modality, those with a minoritized sexual orientation, and Hispanic or Black individuals compared to white participants. The class *interpersonal violence and animal cruelty* reported the highest depression, anxiety, and stress scores, followed by the *interpersonal violence only* group, with the low adversity group reporting the lowest scores (all ps <.001; moderate-to-large effects).

**Conclusion:**

Future research should test whether including animal cruelty in person-centered models of adversity improves identification of high-risk subgroups, and explore how such inclusion can inform multispecies approaches to violence prevention and intervention.

## 1 Introduction

A growing body of research highlights the intersection of animal cruelty and interpersonal violence within family systems (1). Studies have documented that violence toward animals frequently co-occurs with child maltreatment (including emotional, physical, and sexual abuse), as well as children’s exposure to intimate partner violence (1–7). In the context of family violence, cruelty toward animals is not an isolated or random act of harm. Instead, it may be used intentionally as a tactic of coercive control or punishment, contributing to an atmosphere of fear and instability within the household (8–10). For example, in families experiencing intimate partner violence, 50–75% of women report that their partner has harmed or threatened to harm a pet as part of a broader pattern of intimidation, control, and emotional abuse (1).

Children living in such environments may directly witness the intentional harm of companion animals by caregivers or siblings, and witnessing these acts can have attendant psychological consequences (8–11). Exposure to animal cruelty, particularly when the child is emotionally bonded to the pet, has been linked to short and long-term mental health problems (6, 12–14). For instance, Girardi and Pozzulo (15) found that exposure to aggression toward pets in childhood was associated with elevated anxiety and depression symptoms in early adulthood, particularly among those who reported medium levels (compared to low levels) of bonding with their childhood pets. Similarly, Hawkins et al. (16) found that among children aged 7 to 12 years, exposure to animal cruelty amplified the effect of exposure to IPV on generalized internalizing symptoms as well as anxiety, depression, and posttraumatic stress symptoms. In addition to these psychological impacts, childhood exposure to animal cruelty, particularly when it occurs within the broader context of family violence, has been linked to an increased risk of later cruelty toward animals and other forms of antisocial behavior (17–19). These findings underscore the importance of recognizing animal cruelty exposure as both a form of adversity and a potential early marker of broader psychosocial risk.

Although exposure to animal cruelty may contribute to children’s psychological distress and long-term wellbeing (15, 20), it remains absent from most standardized assessments of childhood adversity. Thus, it is not included in widely used instruments such as the World Health Organization’s Adverse Childhood Experiences – International Questionnaire (20–22) or the original Adverse Childhood Experiences (ACE) questionnaire developed by the CDC (23). This exclusion limits opportunities to systematically detect these experiences and understand their intersection and co-occurrence with other forms of adversity. As a result, even though research has demonstrated that exposure to animal cruelty is often linked with other adverse childhood experiences and concomitant health outcomes, it continues to be overlooked in child welfare and population health research (1). Notably, the United Nations Committee on the Rights of the Child recently recognized in General Comment No. 26 (24) that children should be protected from witnessing violence toward animals, affirming the developmental risks posed by such exposure and underscoring the need for its inclusion in child rights and protection frameworks (24).

### 1.1 Exposure to animal harm as threat-based adversity

Recent theoretical developments in childhood adversity research underscore the importance of distinguishing among qualitatively different dimensions of early adversity. The Dimensional Model of Adversity and Psychopathology (25) proposes that distinct dimensions—such as threat (e.g., experiences involving harm or the threat of harm) and deprivation (e.g., absence of expected cognitive and social inputs)—confer risk for psychopathology through different neurodevelopmental mechanisms. Threat-based adversities, including physical and sexual abuse, emotional abuse, and exposure to interpersonal violence, have been linked to altered fear learning, heightened emotional reactivity, and dysregulation of biological stress systems (26–28). Although this model has largely been applied to interpersonal harm, exposure to animal cruelty, particularly when it involves closely bonded animals considered family members, may function similarly (6, 7, 16). Witnessing or learning about violence toward a companion animal can elicit anticipatory fear, helplessness, and relational distress, which are core features of threat-based experiences (6, 25).

From a developmental and biopsychosocial perspective, such experiences may disrupt multiple systems central to emotional regulation and relational security. Because childhood exposure to animal cruelty often occurs within broader contexts of family or community violence that normalize interpersonal harm and undermine safety, empathy, and moral agency, this exposure may further reinforce maladaptive fear learning and anticipatory threat responses (6, 7, 11, 13, 16, 29). Biologically, repeated exposure to animal cruelty and concomitant forms of violence may sensitize stress-response systems such as the hypothalamic–pituitary–adrenal (HPA) axis, contributing to chronic hyperarousal and maladaptive fear learning (25, 30–32). Psychologically, witnessing or losing a bonded companion animal due to harm could evoke traumatic grief and guilt, reinforce internalized helplessness, and impair attachment security (6, 7, 11, 13, 16, 33, 34). Collectively, these pathways illustrate how animal cruelty exposure may operate as a form of threat-based adversity that heightens vulnerability to internalizing distress, particularly when the harm involves a companion animal to which the child is bonded.

### 1.2 Profiles of childhood adversity

Research using person-centered approaches demonstrates that patterns of childhood adversity often cluster in distinct and meaningful ways (35–37). Latent class analysis (LCA), in particular, has been used to identify subgroups of individuals with shared adversity profiles (38). For example, in a community-based sample of young adults, Shin et al. (36) identified four latent classes of ACE exposure patterns (Low ACEs, Household Dysfunction/Community Violence, Emotional ACEs, and High/Multiple ACEs). Accounting for socioeconomic status and gender, they found that individuals in the High/Multiple ACEs class reported significantly higher levels of psychological difficulties. Similarly, in a nationally representative sample of U.S. older adults (37), Kim et al. (37) identified four distinct ACE profiles (Low Adversity, High Adversity, Child Abuse, and Parental Substance Use). Membership in the High Adversity and Child Abuse classes was associated with elevated risk for poor mental health outcomes in later life. These results, along with other studies, suggest that distinct adversity profiles are differentially associated with adult outcomes including depression, anxiety, posttraumatic stress symptoms, substance use, emotion regulation difficulties, and disrupted interpersonal functioning (36, 37, 39, 40).

Although variable-centered studies have demonstrated associations between exposure to animal cruelty and other forms of adversity, person-centered approaches have not yet considered animal cruelty within the broader classification of adverse childhood experiences. As a result, it remains unclear how exposure to this form of violence clusters with other types of adversity in childhood, what contextual factors are associated with its occurrence, and how it may relate to long-term psychological and relational outcomes. This omission is notable, particularly in light of recent evidence demonstrating that animal cruelty is not only prevalent in family violence contexts but may operate as a form of trauma with distinct psychological consequences (6, 7, 13, 16). Understanding these associations is especially important given the central role that companion animals often play in children’s emotional lives and the potential for these experiences to shape later wellbeing (41).

### 1.3 Current study

The present study applied a person-centered approach to examine how exposure to animal cruelty co-occurs with interpersonal threat-based adversity in childhood. Using LCA, we sought to identify distinct profiles of threat-related adversities that include both interpersonal abuse and animal cruelty. Perceived social support and other sociodemographic correlates were included as covariates, given extensive evidence that supportive relationships are associated with improved mental health following adversity and that demographic characteristics such as age, gender, sexual orientation, race and ethnicity are related to both adversity exposure and psychological outcomes (42–47). Guided by the dimensional model of adversity and psychopathology (25), we hypothesized that animal cruelty would cluster with other forms of interpersonal adversity, reflecting its role as a threat-related experience. We further hypothesized that classes characterized by high levels of both interpersonal abuse and animal cruelty would be associated with greater psychosocial difficulties in adulthood compared to low-adversity classes.

## 2 Methods

### 2.1 Participants and procedure

Data were collected from 1,147 U.S. adults aged 18 years or older who participated in the Pets, Attachment, and Mental Health Study, an online survey administered between 6/2/25 and 6/3/25. This study was designed to examine relationships among human–animal attachment, childhood experiences, and adult mental health across a demographically diverse sample of U.S. adults. Participants completed standardized measures of ACEs, pet-related experiences, attachment, and psychological functioning.

The national sample was recruited through Prolific, a licensed online participant recruitment firm that maintains panels of pre-screened participants. Prolific uses verified demographic screening and quota sampling to approximate U.S. census distributions on age, gender, and ethnicity, and its representative-sample option draws participants to match these targets. To ensure data quality, Prolific employs multiple verification procedures (e.g., IP and device fingerprinting, CAPTCHA checks, and periodic attention-screening) and removes accounts that fail quality audits or show fraudulent activity. Peer-reviewed comparisons have shown that Prolific responses demonstrate higher data quality and/or lower rates of inattentive or random responding than other online platforms such as Amazon Mechanical Turk or CrowdFlower (41).

Informed consent was obtained on the first page of the online survey. Participants were provided with information about the study and asked to indicate their willingness to participate before proceeding. The Prolific study description and consent form informed participants that the survey included questions about potentially sensitive or distressing experiences so they could make an informed decision about participation. Participants were paid, via the Prolific platform, $7.50 for completion of the survey and up to an additional $3.00 to complete the pet-related survey items. The present study utilized a subset of variables from the broader survey, which included multiple constructs related to human–animal interaction, early-life experiences, and wellbeing. To minimize priming and social-desirability bias, participants were informed that the survey focused on “childhood and adult experiences, including relationships with pets,” rather than specifically on ACEs or trauma outcomes. Items assessing childhood experiences were embedded among other measures of attachment and wellbeing. All surveys were administered in English. This research was approved by the Institutional Review Board at Colorado State University.

### 2.2 Measures

#### 2.2.1 Adverse childhood experiences

ACEs were assessed using items from the World Health Organization’s ACE-International Questionnaire (21). Eleven items assessed four categories of adversity experienced before the age of 18: 1) Emotional abuse (e.g., being yelled at, insulted, or threatened with abandonment), 2) Physical abuse (e.g., being slapped, kicked, or hit with an object), 3) Sexual abuse (e.g., unwanted sexual touching or intercourse), and 4) Exposure to domestic violence (e.g., witnessing yelling or physical assault between household members). Participants indicated the frequency of exposure to each of the 11 items on a five-point scale ranging from never to always. For the current study, each item was dichotomized to indicate whether the participant was exposed (=1, *rarely, sometimes, most of the time, always*) or not exposed (=0, *never*). If any of the dichotomous items corresponding to the four types of ACEs were endorsed, then that exposure was also represented in the four types of ACEs (1=exposed), whereas if all items for the corresponding type of ACE *never* occurred, the score would be 0 (see **Table 1**).

**Table 1:** Descriptive statistics (N = 1072).

Variables	*M* / #	*SD* / %
Age (in years)	46.36	16.15
Gender identity		
Cisgender man	494	46.1%
Cisgender woman	517	48.2%
Gender minority (transgender woman, transgender man, nonbinary, genderqueer, agender, bigender, genderfluid, two-spirit)	61	5.7%
Sexual orientation		
Heterosexual/Straight	894	83.4%
Sexual minority (bisexual, pansexual, lesbian, asexual, gay, demisexual, queer)	178	16.6%
Race/Ethnicity		
White/non-Hispanic	671	62.6%
Hispanic	130	12.1%
Black/non-Hispanic	130	12.1%
Other race (Asian, biracial/multiracial, Middle Eastern, Native American/Indigenous/American Indian, Native Hawaiian / Pacific Islander)	141	13.2%
Relationship status		
Single, not in a relationship	441	41.1%
In a relationship (married/partnered, engaged, cohabitating, etc.)	631	58.9%
Lived with a pet during childhood		
No	151	14.1%
Yes	921	85.9%
Currently living with a pet		
No	159	14.8%
Yes	913	85.2%
Type of adverse childhood experience (endorsed)		
Emotional abuse	783	73.0%
Physical abuse	700	65.3%
Sexual abuse	347	32.4%
Exposure to domestic violence	705	65.8%
Exposure to animal cruelty	302	28.2%
Mental health severity		
Depression	9.10	10.35
Anxiety	7.62	8.79
Stress	10.31	9.29

#### 2.2.2 Childhood exposure to animal cruelty

Two additional items assessing forms of childhood exposure to animal cruelty (i.e., *Did you see or hear a parent, guardian, or other adult household member hurt a pet on purpose? Did you see or hear a sibling or another child in your household hurt a pet on purpose*)*?* were added for the purposes of this study and are not part of the original ACE-IQ. These items were adapted from the Pet Treatment Survey (48). Each item was rated on a five-point Likert scale from *Never* to *Always*. To align with the scoring of the four types of ACEs, items were dichotomized to reflect whether participants had ever been exposed to either form of animal cruelty (0 = Never, 1 = Ever).

#### 2.2.3 Depression, anxiety, and stress

We assessed depression, anxiety, and stress using the Depression, Anxiety, and Stress Scale–21 (DASS-21 (49, 50);. Each of the DASS-21 subscales included 7 items that indicate the frequency of symptoms as experienced over the past week. The items were scored on a four-point scale ranging from “never” to “almost always.” We calculated subscale scores for depression, anxiety, and stress by summing the item scores and then multiplying them by two for each subscale. This allows for subscale scores to be compared to established cut-off scores indicating the severity of symptoms. Conventional scoring of the DASS-21 indicates that depression, anxiety, and stress scores are considered to be within a normal severity range if they fall between 0-9, 0-7, and 0-14, respectively. Mild scores range from 10-13, 8-9, and 15-18; moderate scores range from 14-20, 10-14, and 19-25; severe scores range from 21-27, 15-19, and 26-33; and extremely severe scores are greater than or equal to 28, 20, and 34, respectively (49). Internal consistency in the current study was good for depression (ω = 0.96), anxiety (ω = 0.94), and stress (ω = 0.94).

#### 2.2.4 Covariates

##### 2.2.4.1 Social support

Perceived social support was assessed using the Multidimensional Scale of Perceived Social Support (51). The MSPSS includes 12 items about whether the participant perceives available support from significant others, family, and friends. Items are ranked on a seven-point scale ranging from “very strongly disagree” to “very strongly agree.” We used the total perceived social support score, which is the mean of responses to all 12 items in this study. Internal consistency of the MSPSS total score was good (ω=0.95).

##### 2.2.4.2 Sociodemographic correlates

Age (continuous), gender modality, sexual orientation, race, ethnicity, relationship status, perceived social support (continuous), childhood pet ownership and current pet ownership were included as sociodemographic covariates. Based on theoretical conceptualizations, we examined whether covariates were associated with latent class membership and with differences in stress, anxiety, and/or depression scores. Gender modality was dummy-coded into three groups: cisgender man, cisgender woman, and gender minority, with cisgender man as the reference group. Sexual orientation was dummy-coded into heterosexual and sexual minority with heterosexual as the reference group. Race and ethnicity were recoded into four dummy variables to address small cell sizes and ensure sufficient statistical power: white/non-Hispanic, Hispanic, Black/non-Hispanic, and another race group (including those who selected Asian, biracial/multiracial, Middle Eastern, Native American/Indigenous/American Indian, or Native Hawaiian/Pacific Islander; see the Discussion section for a review of the limitations of this approach). White/non-Hispanic was used as the reference group. Relationship status, lived with a pet during childhood, and currently living with a pet were dichotomized to indicate whether participants were single/not in a relationship (=0, reference group) or in a relationship (=1, married, partnered, engaged, cohabitating, etc.), did (=1) or did not live with a pet in childhood (=0, reference group), and currently did (=1) or did not live with a pet (=0, reference group).

### 2.3 Analytic strategy

Data cleaning and assumption checks were conducted in SPSS version 30. All other analyses were conducted in Mplus version 8.10. Checks for multicollinearity for the dependent variables were satisfied, as all VIF values were < 10 and conditional index values were < 15. Missing data was minimal (less than 1%) and handled using full information maximum likelihood (FIML) estimation. To avoid having different sample sizes for the LCA and the regression analyses, we conducted analyses with an analytic sample of 1,072 participants. This removed participants who were missing on all of the ACE indicators (*n* = 11, <1% missing) and participants who were missing on any predictor (*n* = 75), which would result in them being excluded from the LCA and/or regression analyses, respectively. Participants who were missing on DASS (*n* = 11) were the same participants missing on all ACE indicators. Most of the missing data was due to missing on any predictor. Examining missingness on each of the predictors separately, 30 participants were missing on race/ethnicity, 21 were missing on current pet ownership, 21 were missing on childhood pet ownership, 20 were missing on gender identity, 17 were missing on sexual orientation, 16 were missing on relationship status, four participants were missing on age, and one was missing on perceived social support. Missingness on these variables was negligible (<3%). We examined descriptive statistics for the ACEs indicators, covariates, additional sociodemographic variables, and DASS-21 subscales. Bivariate correlations were tested for the DASS-21 subscales.

We conducted latent class analysis to identify unobserved subgroups based on participants’ adversity profiles (52). Models with one to six classes were estimated. We evaluated model fit using standard criteria, including the Akaike Information Criterion (AIC), Bayesian Information Criterion (BIC), sample-size adjusted BIC (SABIC), likelihood ratio tests (Lo–Mendell–Rubin (LMR) LRT, Vuong–Lo–Mendell–Rubin (VLMR) LRT), and bootstrap LRT (BLRT). Lower values on the AIC, BIC, and SABIC indicate better model fit. The LRTs test whether the additional model complexity of a *k* + 1 class is a significant improvement in model fit compared to a *k* class model, with a significant *p*-value indicating significant improvement in fit and the *k* + 1 class should be retained. Additional considerations included entropy, average posterior probabilities (AvePP), minimum class size, and interpretability of the resulting profiles. Entropy greater than 0.80 is an indication of “good” classification of individuals into latent classes and AvePP greater than 0.70 is indicative of good class separation (53, 54). We compared potential candidate models (e.g., three-, four-, and five-class solutions) using the item probabilities, class sizes, and interpretability of the subgroups.

Next, to examine subgroup differences in covariates, depression, anxiety, and stress, we used the Bolck-Croon-Hagenaars (BCH) approach (55). This approach is recommended as it allows for the examination of covariates and distal outcomes in the same model while preventing the latent classes from shifting while accounting for individual classification error. The first step of the BCH approach creates BCH weights that account for classification error into subgroups. The final step of the BCH approach allows for the testing of covariates and distal outcomes. To test for subgroup differences based on covariates, we regressed the latent class variable on the covariates (age, gender modality, sexual orientation, race/ethnicity, and living with a pet during childhood) and used Wald tests to determine the significance of each association, separately. Finally, we used Wald tests to evaluate whether there were significant mean differences in depression, anxiety, and stress across subgroups, adjusting for covariates (age, gender modality, sexual orientation, currently living with a pet, relationship status, social support, and race/ethnicity). We explored more specific pair-wise differences using the model constraint function in Mplus.

## 3 Results

### 3.1 Univariate and bivariate statistics

The most frequently endorsed type of ACE was emotional abuse (73.0%), followed by exposure to domestic violence (65.8%) and physical abuse (65.3%). Less frequently endorsed were sexual abuse (32.4%) and exposure to animal cruelty (28.2%). The dependent variables, depression, anxiety, and stress, were all positively correlated. Depression was moderately to strongly associated with anxiety (*r* = 0.72, *p* <.001) and stress (*r* = 0.78, *p* <.001), and anxiety was strongly associated with stress (*r* = 0.81, *p* <.001). Although the average score for stress was within the normal range (*M* = 10.31, *SD* = 9.29), the scores of depression (*M* = 9.10, *SD* = 10.35) and anxiety (*M* = 7.62, *SD* = 8.79) indicated that the average scores in this sample were mild in severity.

### 3.2 Latent class enumeration

The LCA produced models with one to six latent classes. The fit statistics across these models suggested that the three-class model fit our data best (see **Table 2**). The AIC, BIC, and SABIC were all lowest in the three-class model. The LRTs also provide support for this model, as the three-class model was a significant improvement in fit compared to the two-class model, but the added complexity of the four-class model did not significantly improve fit above and beyond the three-class model. Although less than ideal, the entropy of the three-class model was adequate (entropy = 0.72). Further, the average posterior probabilities ranged from 0.71 to 0.91, which indicate that there is adequate separation between classes and precision.

**Table 2:** Fit indices for unconditional latent class models with 1-7 classes.

*k*	Par	LL	AIC	BIC	SABIC	VLMR-LRT p-value	LMR-LRT p-value	BLRT p-value	Entropy	Condition #	Smallest n
1	5	-3318.1	6646.1	6671.0	6655.1					1.30E-01	100%
2	11	-2724.8	5471.5	5526.3	5491.3	<.001	<.001	<.001	.824	2.68E-02	35.7%
**3**	**17**	**-2651.0**	**5336.0**	**5420.6**	**5366.6**	**<.001**	**<.001**	**<.001**	**.715**	**3.96E-03**	**29.6%**
4	23	-2646.3	5338.5	5453.0	5379.9	.348	.356	.214	.768	2.76E-06	3.17%
5	29	-2644.1	5346.2	5490.5	5398.4	.289	.295	.429	.848	1.30E-06	1.9%
6	35	-2643.3	5356.5	5530.7	5419.6	.751	.755	.667	.813	3.58E-07	0.8%
7	Model did not converge										

*N* = 1,072; *k* = number of classes, Par = number of parameters, LL = log likelihood, AIC = Akaike information criterion, BIC = Bayesian information criterion, SABIC = sample-size adjusted BIC, VLMR-LRT = Vuong-Lu-Mendell-Rubin likelihood ratio test, LMR-LRT = Lu-Mendell-Rubin likelihood ratio test, BLRT = bootstrapped likelihood ratio test. The bolded values indicate the selected model.

**Figure 1:**
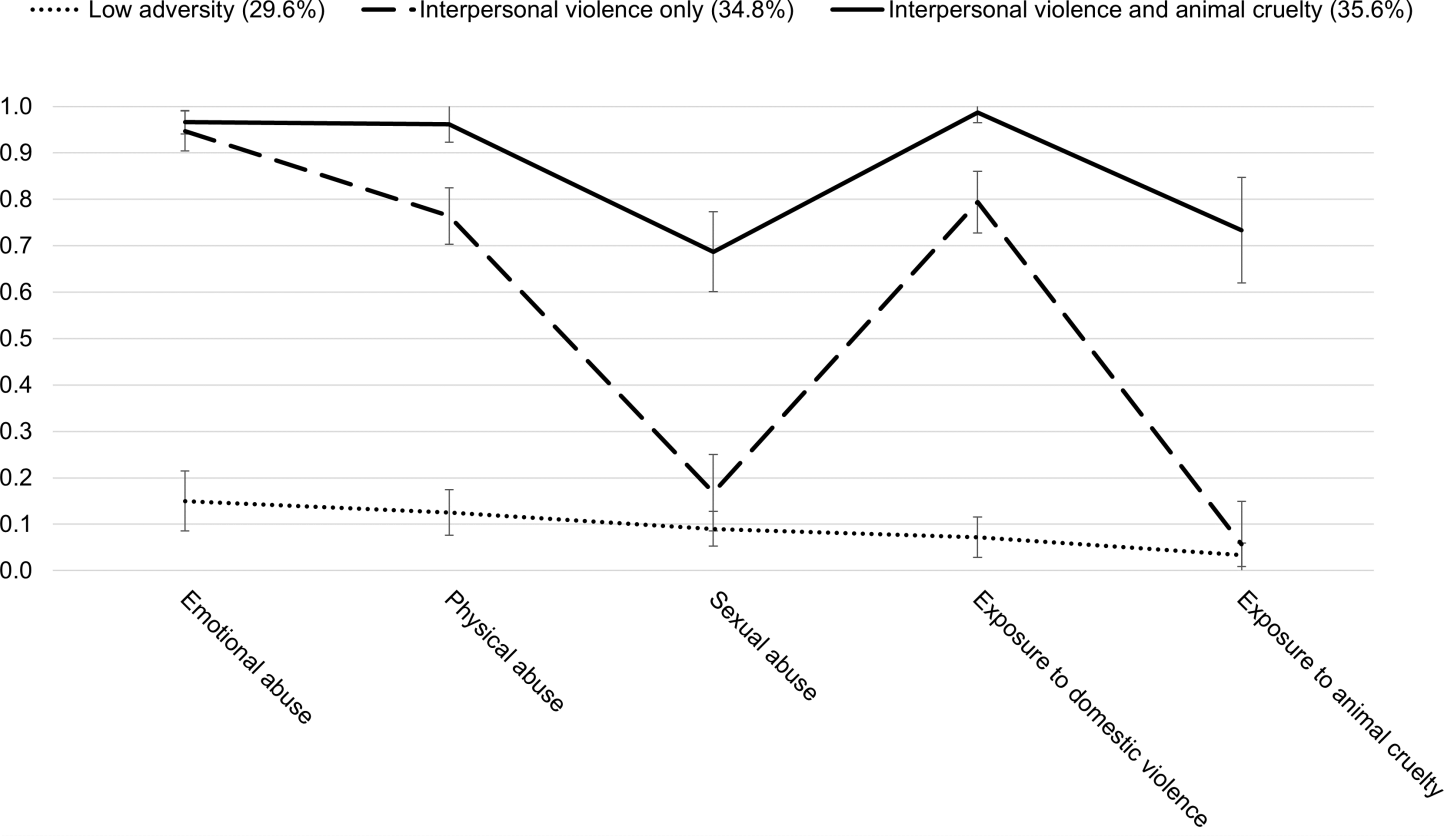
Item probability plot for the 3-class model (N = 1,072). The error bars represent 95% confidence intervals.

We used the probability of endorsing each of the five types of ACEs to label the three subgroups (see **Figure 1**). The first subgroup contained 29.6% of the sample and was characterized by low probability (< 0.30) of endorsing all five types of adversity. Therefore, we labeled this subgroup the *low adversity* subgroup. The second subgroup was labeled the *interpersonal violence only* subgroup (34.8%) as it was characterized by high probability (> 0.70) of endorsing emotional abuse, physical abuse, and exposure to domestic violence and low probability of endorsing sexual abuse and exposure to animal cruelty. The last subgroup (35.6%) was labeled *interpersonal violence and animal cruelty* as this group had high probability of endorsing all of the following: emotional abuse, physical abuse, sexual abuse, exposure to domestic violence, and exposure to animal cruelty. We included sexual abuse endorsement as the probability in this subgroup was 0.69, which we considered close enough to the cut-off to be characteristic exposure.

### 3.3 Sensitivity analysis

Although not included in the current study, we also conducted a sensitivity analysis of the latent class enumeration process using the ordinal frequency response scales (never, rarely/sometimes, most of the time/always). These results did not provide clear defining patterns of frequency of ACE exposures, and thus models using the ordinal response scale were not carried forward. We provide further detail in the **Supplementary Materials**.

### 3.4 Differences in subgroup membership by sociodemographic characteristics

Our comparisons of posterior class probabilities indicated significant differences in subgroup membership based on age, gender modality, sexual orientation, and race/ethnicity (see **Table 3**). However, living with a pet during childhood was not significantly associated with subgroup membership, x^2^(2) = 3.47, *p* = .177. Age was significantly associated with subgroup membership, x^2^(2) = 8.00, *p* = .018. Specifically, compared to membership in the *low adversity* subgroup, as age increased, the likelihood of being in the *interpersonal violence and animal cruelty* subgroup decreased (*OR* = 0.98, 95% CI: 0.97, 0.99). There was no significant association between membership in the *interpersonal violence only* subgroup compared with the *low adversity* subgroup based on age.

**Table 3:** Associations between covariates and latent classes and between covariates and stress, anxiety, and depression by latent class membership.

Variable	Class 1 (reference class)		Class 2		Class 3	
	Estimate	*P-*value	Estimate	*P-*value	Estimate	*P-*value
Age	–	.	-0.01	.295	-0.02	.005
Sexual Orientation (= sexual minority)	–	–	0.14	.683	0.98	.001
Childhood Pet (= yes)	–	–	0.37	.200	-0.23	.380
Cis-gender woman	–	–	0.001	.996	0.34	.074
Gender minority	–	–	0.22	.793	**1.68**	**.005**
Hispanic	–	–	-0.04	.900	0.16	.568
Black	–	–	0.12	.733	**0.94**	**.001**
Other race	–	–	0.36	.173	-0.70	.052

Depression						
Variable	Class 1		Class 2		Class 3	
	β	*P-*value	β	*P-*value	β	*P-*value
Age	**-0.13**	**<.001**	**-0.12**	**<.001**	**-0.12**	**<.001**
Sexual orientation (= sexual minority)	0.01	.651	0.01	.652	0.02	.653
Current pet (= yes)	0.02	.503	0.02	.503	0.01	.503
Relationship status (= in a relationship)	0.02	.635	0.02	.635	0.01	.635
Perceived social support	**-0.41**	**<.001**	**-0.46**	**<.001**	**-0.44**	**<.001**
Cisgender woman	-0.03	.289	-0.03	.289	-0.03	.290
Gender minority	0.04	.065	0.05	.063	0.10	.062
Hispanic	-0.004	.868	-0.004	.868	-0.004	.868
Black	-0.03	.205	-0.03	.203	-0.04	.204
Other race	-0.03	.212	-0.03	.213	-0.02	.214

Anxiety						
Variable	Class 1		Class 2		Class 3	
	β	*P-*value	β	*P-*value	β	*P-*value
Age	**-0.20**	**<.001**	**-0.21**	**<.001**	**-0.18**	**<.001**
Sexual orientation (= sexual minority)	-0.02	.485	-0.03	.485	-0.03	.484
Current pet (= yes)	0.06	.065	0.06	.066	0.04	.071
Relationship status (= in a relationship)	0.09	.017	0.09	.014	0.07	.019
Perceived social support	**-0.16**	**<.001**	**-0.19**	**<.001**	**-0.17**	**<.001**
Cisgender woman	-0.03	.403	-0.03	.405	-0.03	.405
Gender minority	**0.06**	**.038**	**0.07**	**.035**	**0.13**	**.034**
Hispanic	-0.01	.868	-0.01	.869	-0.01	.868
Black	-0.04	.202	-0.04	.206	-0.05	.203
Other race	-0.04	.211	-0.05	.212	-0.03	.213

Stress						
Variable	Class 1		Class 2		Class 3	
	β	*P-*value	β	*P-*value	β	*P-*value
Age	**-0.20**	**<.001**	**-0.19**	**<.001**	**-0.19**	**<.001**
Sexual orientation (= sexual minority)	0.01	.742	0.01	.742	0.02	.742
Current pet (= yes)	0.02	.534	0.02	.534	0.01	.534
Relationship status (= in a relationship)	**0.08**	**.017**	**0.08**	**.018**	**0.07**	**.017**
Perceived social support	**-0.23**	**<.001**	**-0.26**	**<.001**	**-0.26**	**<.001**
Cisgender woman	0.01	.885	0.01	.885	0.004	.885
Gender minority	**0.06**	**.009**	**0.07**	**.010**	**0.15**	**.008**
Hispanic	-0.01	.868	-0.004	.868	-0.01	.868
Black	-0.04	.206	-0.03	.205	-0.05	.204
Other race	-0.04	.212	-0.04	.213	-0.03	.212

Bolded estimates indicate statistically significant associations. Regression coefficient estimates of the association between covariates and latent class membership are unstandardized; however, regression coefficient estimates of the association between covariates and depression, anxiety, and stress by class are standardized. Gender identity and race/ethnicity were dummy coded, such that cisgender man and White/non-Hispanic were the reference groups, respectively.

There were significant differences in subgroup membership based on gender modality, x^2^(4) = 12.81, *p* = .012. Cisgender men and cisgender women had higher odds of being in the *low adversity* subgroup (*OR* = 3.28, *p* <.001 and *OR* = 2.73, *p* = .001, respectively) and the *interpersonal violence only* subgroup (*OR* = 2.45 and 2.06, *p*s <.001, respectively) compared to those with a minoritized gender modality. In contrast, individuals with a minoritized gender modality were more likely to be in the *interpersonal violence and animal cruelty* subgroup compared to cisgender men (*OR* = 4.79, *p* <.001) and cisgender women (*OR* = 3.40, *p* <.001).

We also found significant differences in subgroup membership based on sexual orientation, x^2^(2) = 14.45, *p* = .001. Individuals with a minoritized sexual orientation had higher odds of being in the *interpersonal violence and animal cruelty* subgroup, relative to heterosexual individuals (*OR* = 2.50, *p* <.001). In contrast, the odds of being in the *interpersonal violence only* (OR = 0.64, *p* = .001) and *low adversity* subgroups (*OR* = 0.53, p <.001) were lower for individuals who held minoritized sexual identities in comparison to heterosexual individuals.

There were also significant differences in subgroup membership based on race/ethnicity, x^2^(6) = 22.11, *p* = .001. Relative to white individuals, Hispanic and Black individuals had lower odds of being in the *low adversity* subgroup (*OR* = 0.94, p = .001 and OR = 0.54, *p* = .002, respectively) and the *interpersonal violence only* subgroup (*OR* = 0.89, *p* = .007 and *OR* = 0.65, *p* = .012, respectively). Individuals in the other racial/ethnic identity group had higher odds of being in the *low adversity* subgroup (*OR* = 1.03, *p* = .001) and the *interpersonal violence only* subgroup (*OR* = 1.88, *p* = .004) compared to white individuals. The pattern reversed for the *interpersonal violence and animal cruelty* subgroup. Hispanic (*OR* = 1.19, *p* = .003) and Black individuals (*OR* = 2.42, *p* = .002) had higher odds of being in this subgroup compared to white individuals. Those with another racial/ethnic identity had lower odds of being in this subgroup compared to white individuals (*OR* = 0.42, *p* = .015).

### 3.5 Associations between covariates and depression, anxiety, and stress

Across classes, age was negatively associated with depression (*βs* range: -0.12 to -0.13, *ps* <.001), anxiety (*βs* range: -0.18 to -0.21, *ps* <.001), and stress (*βs* range: -0.19 to -0.20, *ps* <.001). Similarly, social support was negatively associated with depression (*βs* range: -0.41 to -0.46, *p*s <.001), anxiety (*βs* range: -0.16 to -0.19, *p*s <.001), and stress (*βs* range: -0.23 to -0.26, *p*s <.001) across all classes. On average, individuals who were in a relationship reported higher stress (*βs* range: 0.07 to 0.08, *ps <*.05) and anxiety (*βs* range: 0.07 to 0.09, *ps <*.05) across classes compared to those who were single. Those with a minoritized gender modality had higher anxiety across classes (*βs* range: 0.06 to 0.13, *ps <*.05) and higher stress across classes (*βs* range: 0.06 to 0.15, *ps <*.05) compared to cisgender men. No other associations between predictors and outcome variables were significant (see **Table 3**).

### 3.6 Differences in depression, anxiety, and stress across classes

**Figure 2:**
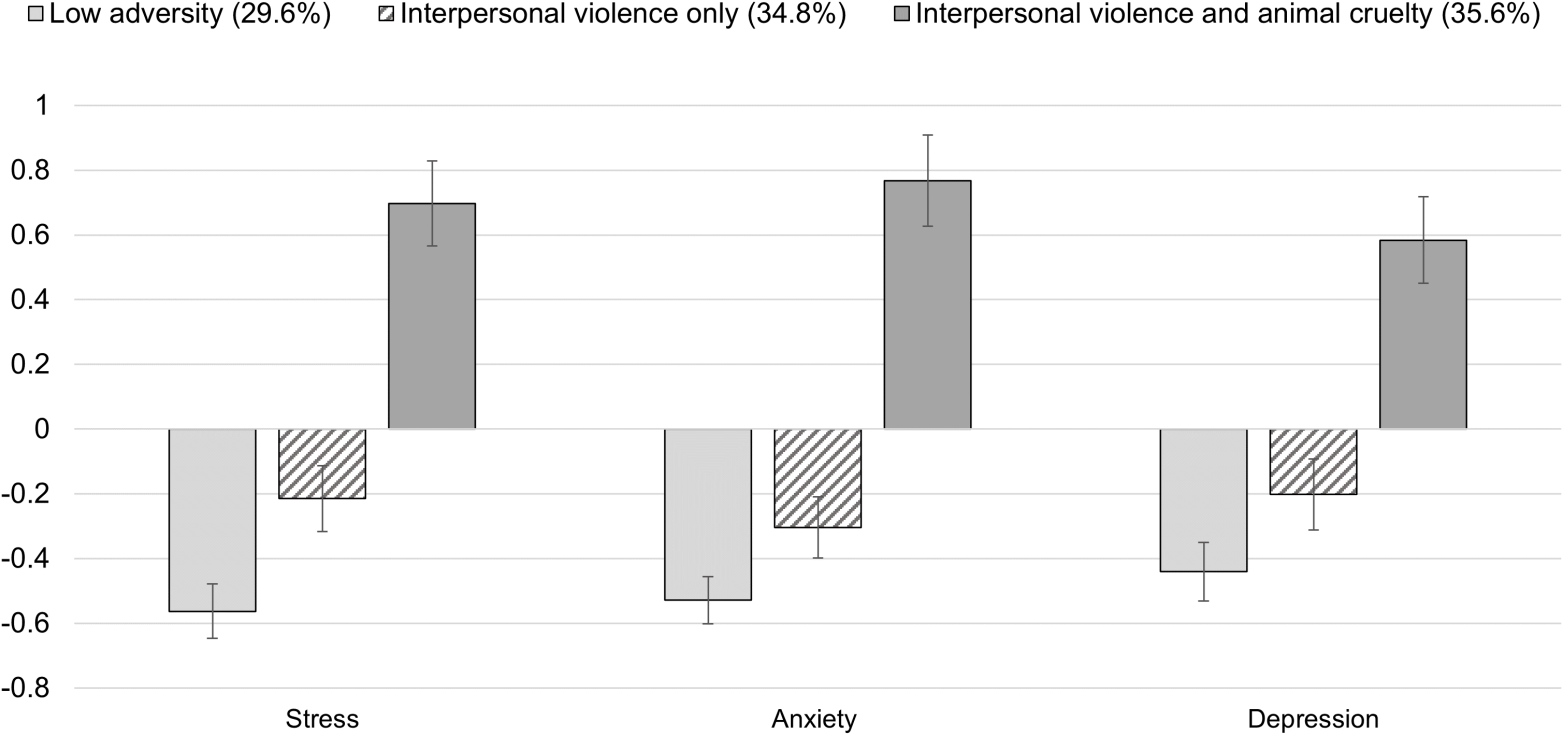
Mean Scores of Depression, Anxiety, and Stress Across Subgroups (N = 1,072). Y-axis values represent standard deviations from the sample mean. Error bars represent 95% confidence intervals.

There were significant differences in mean scores for depression, anxiety, and stress across classes. **Figure 2** displays the standardized means of depression, anxiety, and stress. There were significant differences across classes in depression scores, x^2^(2) = 75.43, *p* <.001. The *low adversity* and the *interpersonal violence only* classes reported lower depression scores than those in the *interpersonal and animal cruelty* class (*d* = 0.73 and *d* = 0.61, *ps* <.001, respectively). There was no significant difference in depression scores between the *low adversity* and the *interpersonal violence only* classes (*d* = 0.12, *p* = .075).

All classes differed significantly from each other in their anxiety scores, x^2^(2) = 162.95, *p* <.001. The *low adversity* class had lower anxiety scores compared with the *interpersonal only* (*d* = 0.19, *p* = .003) and the *interpersonal violence and animal cruelty* classes (*d* = 1.13, *p* <.001). The *interpersonal violence and animal cruelty* class reported higher anxiety scores than the *interpersonal violence only* class (*d* = 0.95, *p* <.001).

Lastly, classes differed in their stress scores, x^2^(2) = 150.82, *p* <.001. All classes differed significantly from one another in the anticipated directions. The *low adversity* class had lower stress scores than the *interpersonal violence only* (*d* = 0.28, *p* <.001) and the *interpersonal violence and animal cruelty* classes (*d* = 1.03, *p* <.001). The *interpersonal violence and animal cruelty* class also reported higher stress scores than the *interpersonal violence only* class (*d* = 0.75, *p* <.001).

## 4 Discussion

This study extends the ACEs literature by integrating childhood exposure to animal cruelty into a person-centered analysis of threat-based adversities. Using LCA, we identified three distinct classes: *low adversity, interpersonal violence only*, and *interpersonal violence with animal cruelty*. The emergence of a class characterized by high co-occurrence of human- and animal-directed violence underscores the importance of applying a multispecies lens to the study of childhood adversity and developmental risk, particularly given prior research suggesting that exposure to animal cruelty is associated with compromised socioemotional functioning among children and adults (7, 16, 56, 57). Furthermore, our findings correspond to the threat dimension within the Dimensional Model of Childhood Adversity which frames experiences involving harm or threat of harm as conferring risk for psychopathology through mechanisms such as increased emotional reactivity and dysregulation of stress-response systems (25).

In our sample, 28.2% of participants reported childhood exposure to animal cruelty. This aligns with the lower range of prevalence estimates from U.S. samples where children are affected by intimate partner violence, in which studies have reported that 25–57% of children witness threats or harm to companion animals (1, 2, 6, 58). In contrast, general population and college samples show markedly lower rates. For example, Carlisle-Frank et al. (59) found that only 4% of college students recalled witnessing harm to a pet, and similar single-digit rates have been documented among broader community samples (8, 17, 60). Our findings suggest that animal cruelty exposure, while not ubiquitous, is elevated in settings of complex family violence and that prevalence may vary across population subgroups, such as those defined by demographic factors like age.

Notably, our rates for other adversities—emotional abuse (73%), physical abuse (65%), sexual abuse (32%), and witnessing domestic violence (66%)—are substantially higher than those observed in general U.S. adult populations. These elevated rates likely reflect differences in item wording and sampling approach. Compared to single-item ACE measures used in surveillance studies, our multi-item indicators may have lowered the threshold for endorsement, while online, self-selected participation through Prolific could increase willingness to disclose sensitive experiences. Data from CDC’s Behavioral Risk Factor Surveillance System indicate much lower prevalence, with estimates around 34-35% for emotional abuse, 16–23% for physical abuse, 11-13% for sexual abuse, and 15-17% for witnessing domestic violence (47, 61, 62). Other large-scale studies, such as Felitti et al. (23) and Ports et al. (63), similarly report lower base rates (63, 64). In our study, emotional abuse included an item for being “yelled, screamed, or sworn at,” which may have resulted in more endorsements than the ACE study item “swear at, insult, or put you down.” Similarly, physical abuse included an item for having been “spanked, slapped, kicked, punched, or beat up,” whereas the ACE study item includes “hit, beat, kick, or physically hurt you.” The inclusion of spanking as an indicator of physical abuse may have also lowered the threshold for this type of adversity, as rates of spanking in the U.S. are as high as 49% (65).

Discrepancy in measurement and differences in samples have been shown to result in disparate rates of reporting of emotional abuse. [e.g., 6.9% vs. 83% (66)]. High rates of emotional, physical, and sexual abuse and exposure to domestic violence similar to ours have been found among low-income mothers (60%, 42.4%, 43.5%, 46.4%, respectively (67)). There is also evidence that rates of ACEs have increased over the course of 14 years [2009-2022 (68)], which may indicate a trend in greater recognition of ACEs and of certain parenting behaviors (e.g., spanking) as harmful; this may help to explain why our rates are higher than previous studies. Further, our study relied on a binary assessment of exposure to ACEs (i.e., exposed vs. not exposed), which may over-estimate exposure. For example, in the current sample, emotional abuse and physical abuse endorsement are closer to 50% if those who experienced these *rarely* are not considered exposed. Future studies should take into consideration the frequency of exposure to ACEs and the thresholds of adversity that increase risk for negative mental and physical health outcomes. This study should also be replicated to examine the co-occurrence of animal cruelty with other forms of childhood adversity in samples with potentially different prevalence rates.

The elevated mental health symptoms observed in the interpersonal violence and animal cruelty class are consistent with prior variable-centered studies showing that animal cruelty exposure amplifies the effects of other forms of family violence on psychological distress (15, 16). These findings also align with the Dimensional Model of Adversity and Psychopathology (25), which posits that threat-based adversities (experiences involving harm or the threat of harm) are associated with heightened emotional reactivity, dysregulated stress physiology, and altered fear learning; all processes that may contribute directly to the elevated risk for anxiety, depression, and stress observed in this sample (25, 30–32). Witnessing harm to an animal, particularly one with whom a child has an emotional bond, represents a distinct adversity that can elicit fear, helplessness, grief, and disruption of attachment relationships, while also potentially compounding the psychological impact of other forms of violence or maltreatment. Beyond internalizing difficulties, witnessing or experiencing animal cruelty in childhood has also been linked to externalizing outcomes. Prior work indicates that such exposure is associated with cruelty perpetration, broader antisocial behavior, and later justice-system contact (69); future longitudinal research should test whether ACE profiles that include animal cruelty predict these outcomes above and beyond classes characterized by interpersonal forms of violence alone.

The sociodemographic differences in latent class membership, including the overrepresentation of individuals with minoritized gender identities, sexual orientations, and certain racial/ethnic backgrounds in the *interpersonal violence and animal cruelty* subgroup, parallel prior evidence that marginalized populations face disproportionate exposure to violence (70–72) and systemic barriers to safety and care (73, 74). Although individuals in the “other” racial/ethnic identity group had higher probabilities of being in the low adversity and interpersonal violence only subgroups, interpretation is limited by the heterogeneity of this category. Prior research has documented that some groups, such as Native American individuals, tend to experience higher rates of adversity, whereas others, such as Asian individuals, often report lower rates (75). Grouping these identities together may have obscured meaningful differences in the findings. Future studies should aim to explore these identities separately and with an intersectional lens (e.g., the intersection of race and gender) in order to better ascertain racial/ethnic variation in patterns of ACEs exposure. Although we found that animal cruelty often co-occurred with other forms of violence (76–82), prior research suggests this overlap reflects broader structural risks (e.g., poverty, racism, cumulative adversity) that differentially burden marginalized communities (83–87). This is further corroborated by a study by Reese et al. (85) that found that race, gender, and age were not associated with animal cruelty. Rather, they found that neighborhood conditions in terms of economic stress, vacancy and blight, and crime appear to be most strongly associated with animal cruelty in urban Detroit. Recognizing these patterns underscores the need to interpret the co-occurrence of human- and animal-directed harm in light of social and structural inequities, rather than attributing it to inherent features of individuals or groups.

### 4.1 Implications

From a clinical perspective, these findings emphasize the importance of integrating animal welfare considerations into trauma-informed assessment and intervention. The identification of three distinct adversity classes, and particularly the elevated depression, anxiety, and stress symptoms in the *interpersonal violence and animal cruelty* class, suggests that animal cruelty can signal severe, multifaceted trauma. Clinicians should anticipate complex presentations involving disruptions in trust and attachment, heightened physiological arousal, and grief or guilt tied to harm of companion animals (33). Comprehensive screening protocols should explicitly include questions about animal-directed harm, particularly in contexts where pets are present, to improve detection of complex household violence. Treatment approaches combining evidence-based trauma therapies such as Trauma-Focused Cognitive Behavioral Therapy (TF-CBT) and Eye Movement Desensitization and Reprocessing (EMDR) with interventions addressing grief, guilt, and relational disruptions related to pet loss or harm are recommended (84, 88).

We also recognize ongoing debate regarding the potential harms and unintended consequences of routine ACEs screening. Scholars have cautioned that universal or poorly contextualized screening may lead to stigma, retraumatization, or inadequate follow-up care if it is not paired with appropriate support (89–91). Accordingly, our recommendations align with calls for targeted, trauma-informed assessment rather than broad population-level screening. Within this context, questions about pets and their wellbeing may provide a more relationally sensitive means of understanding family dynamics and safety (6). Pets often function as emotional and relational agents within families, and discussing their wellbeing can surface information about caregiving, attachment, and household stress that individuals may be hesitant to disclose through more direct questioning. Qualitative studies show that children experiencing domestic violence frequently describe pets as sources of comfort or emotional security amid household conflict, and that harm or threats toward animals can reveal underlying coercion or relational distress (6, 8, 92). Integrating pet-related inquiry within trauma-informed frameworks—paired with clear referral pathways and ethical safeguards—may therefore offer clinicians a compassionate, non-confrontational approach to exploring complex family processes. Further research should examine this approach empirically to determine its validity, feasibility, and boundaries, and how it can be embedded within broader cross-sector efforts to identify and respond to multispecies forms of household violence.

At the systems level, cross-sector collaboration between child welfare, domestic violence services, and animal protection organizations can strengthen early detection and coordinated responses (93). Implementation planning should establish feasible cross-reporting pathways, safeguard data-sharing, and provide workforce training that equips professionals across sectors (including veterinary and animal-services staff) to recognize and respond to suspected maltreatment. At the same time, safeguards are essential to avoid disproportionate surveillance of marginalized families, who are already more likely to experience system involvement and punitive responses (94, 95). Clear guidelines, accountability measures, and meaningful community input can help ensure that cross-reporting enhances safety without reinforcing or exacerbating existing inequities (95). These safeguards are particularly important given sociodemographic differences in class membership, which highlight the need for culturally responsive practices. The overrepresentation of individuals with minoritized gender identities, sexual orientations, and racial/ethnic backgrounds in the interpersonal violence and animal cruelty class underscores how systemic discrimination and structural inequities compound exposure to complex household violence (95, 96). To address these inequities, clinicians and service providers should approach assessment with cultural humility, awareness of barriers to safety and care, and readiness to respond to the cumulative impact of interpersonal and systemic trauma. Community-based outreach, training in cultural competence, and co-designed screening protocols can enhance trust, mitigate unintended harms, and increase relevance for the populations most affected (97).

At the prevention and policy levels, education about the co-occurrence of human- and animal-directed violence, stigma reduction around disclosure, and multisector service coordination remain essential (93, 98). A tiered intervention framework that combines universal education with targeted services for higher-risk subgroups may be especially effective. Future research should examine whether safety planning that explicitly includes animal companions and acknowledges multispecies family systems improves engagement and outcomes. Finally, while advocacy groups often highlight “the Link” between animal cruelty and interpersonal violence in broad terms, our findings point to the need for more nuanced, evidence-based communication. In this sample, animal cruelty co-occurred with other adversities only in the highest-risk subgroup, suggesting that its predictive value is context-dependent. Prevention messaging should therefore emphasize the conditions under which animal cruelty is most strongly associated with other forms of violence, rather than overgeneralizing across contexts (34, 93).

Another implication concerns ongoing debates in the ACEs literature regarding construct proliferation (i.e., the ever-expanding list of indicators now included under the ACE umbrella) and the conflation of exposure with symptomatology, which can blur conceptual boundaries and inflate associations with later outcomes (99–101). Some scholars argue that continual ACE expansion risks diluting the construct’s theoretical coherence and utility for prevention and policy, while others emphasize the importance of incorporating *contextually* and *developmentally* salient forms of adversity that have been historically overlooked. Our analytic approach addresses these concerns by restricting LCA indicators to threat-based exposure events and modeling psychopathology (depression, anxiety, and stress) as distal outcomes, maintaining a conceptual separation between adversity and sequelae. Including animal-cruelty exposure thus represents a theory-driven refinement—situating it within the threat dimension of the Dimensional Model of Adversity and Psychopathology rather than as an unbounded expansion of ACEs. We also recommend that future research test model sensitivity to indicator inclusion, avoid undifferentiated ACE “sum scores,” and continue advancing mechanism-focused frameworks that distinguish adversity exposures from their psychological and biological outcomes.

### 4.2 Limitations and future directions

Several limitations warrant consideration. One limitation is that ACEs were assessed using a subset of WHO ACE-IQ items limited to interpersonal violence, supplemented with two animal cruelty items developed for this study. Although this approach aligned with our focus on threat-based adversities, it excluded other important domains, such as neglect and household dysfunction, limiting our ability to capture the full spectrum of adversity. Another limitation is that all data were retrospective and self-reported, which may introduce recall bias and misclassification. Additionally, the cross-sectional design precludes conclusions about temporal ordering or causal relationships between adversity class membership and mental health outcomes.

Although the sample was nationally recruited through Prolific, a paid online research platform that approximates U.S. census distributions on key demographics, several features of the sample may limit generalizability. Most participants reported current (85%) or past (86%) experience with companion animals, which may reduce representativeness for individuals without such experiences. The sample was also predominantly white, cisgender, and heterosexual, potentially constraining generalizability and limiting representation of populations disproportionately affected by adversity, including racially minoritized and LGBTQ+ groups (102–104). These intersecting identities can shape both exposure to adverse experiences and access to supportive relationships—including with animals—through structural inequities such as discrimination, housing insecurity, or barriers to pet ownership. Future research should aim to recruit more diverse samples and explore whether the protective or mediating roles of human–animal relationships differ across cultural contexts and systems of marginalization. As previously discussed, participants who self-select into compensated online studies may also differ from the general population in their comfort disclosing sensitive experiences, and those with strong connections to animals may have been especially motivated to participate, potentially resulting in overrepresentation of pet owners.

A further consideration is that our analytic approach grouped racial and ethnic identities into broad categories due to sample size considerations. While this was necessary for statistical power, it may obscure important differences in adversity exposure and mental health outcomes across more specific racial and ethnic subgroups. Prior research demonstrates meaningful heterogeneity in ACE prevalence and sequelae across diverse populations (47, 105). Future studies should prioritize sufficient representation to allow for disaggregated analyses that more accurately capture the experiences of historically marginalized communities and contains sufficient power for exploring intersecting identities.

Another limitation concerns measurement of animal cruelty; the items did not include contextual features such as severity, frequency, perpetrator relationship, or emotional closeness to the harmed animal, all of which likely shape psychological impact (6, 7, 16). Future measurement work should evaluate item performance of the animal cruelty items alongside other ACEs, assess invariance across sociodemographic groups, and incorporate gradient scoring (e.g., severity, chronicity, relational proximity).

Finally, our assessment of ACEs dichotomized items into ever exposed or never exposed. Although this approach has been used in prior LCA studies (37, 106–108), it may overestimate exposure to ACEs that occurred infrequently in childhood and limit the ability to analyze outcomes based on frequency of exposure. We also chose to include sexual abuse exposure as a defining characteristic of the *interpersonal violence and animal cruelty* subgroup due to a 0.69 probability of endorsement; however, distributions of exposure may differ in other samples due to the sample-specific nature of LCA. Future research should replicate the current study and explore whether classes of individuals based on patterns of frequency of exposure to ACEs can be identified to support the presence of these subgroups in the larger population and to determine whether additional subgroups can be identified.

Future research can build on these findings in several ways. Longitudinal studies are needed to examine whether the adversity profiles identified here predict trajectories of mental health, social functioning, and relational outcomes across developmental stages. Expanding adversity measurement to include both threat- and deprivation-based domains would allow for a more comprehensive understanding of how adversity impacts multispecies households. More nuanced and precise animal cruelty measurement (e.g., severity, frequency, relational context) could also clarify how different features of cruelty exposures contribute to psychological outcomes (13). Additionally, qualitative studies could further illuminate children’s lived experiences of animal cruelty within the ecology of family violence, providing insight into meaning-making, coping, and recovery, and informing intervention strategies. Although prior work has emphasized overlap between intimate partner violence and animal cruelty (6, 11, 16), few studies have examined how animal cruelty interacts with broader constellations of childhood adversity.

A key priority for future research is to test whether ACE profiles that include animal cruelty exposure predict externalizing outcomes (e.g., cruelty perpetration, delinquency, arrests) and multi-system contact (e.g., child welfare, juvenile justice, behavioral health) linked to broader antisocial behavior (2, 109–111). Prospective designs using latent transition analysis (LTA) could examine movement between classes over time and clarify directionality of effects. Incorporating multi-informant data (e.g., caregiver reports, administrative records) would strengthen causal inference, reproducibility, and generalizability. Finally, intervention studies should test whether integrating animal welfare considerations into trauma-informed care, including safety planning for pets and grief-focused supports, improves engagement, safety, and outcomes for survivors of complex household violence. Together, such work would advance our understanding of animal cruelty as a meaningful component of childhood adversity and inform multispecies approaches to prevention, assessment, and intervention.

## 5 Conclusion

Exposure to violence directed toward animals in the home is an important and under-recognized form of childhood adversity. A majority (60%–80%) of U.S. children live with a companion animal (112, 113), and many consider these animals to be members of the family (114). Moreover, a substantial proportion of individuals report witnessing or being aware of harm to animals during childhood (2, 8). By integrating exposure to animal cruelty into a person-centered model of ACEs, this study demonstrates how patterns of violence can span across species boundaries. Understanding these patterns is essential in creating more inclusive models of adversity and developing prevention and intervention strategies that reflect the realities of children’s lives in multispecies families. Our findings underscore the value of incorporating animal cruelty exposure into ACE assessments, advancing translational efforts that align trauma-informed clinical care with coordinated responses across child welfare, domestic violence, and animal-protection systems.

## Data Availability

The datasets generated and analyzed for this study are not publicly available due to participant confidentiality and ethical restrictions under the approved Colorado State University IRB protocol. Qualified researchers may request access to de-identified data from the corresponding author (Dr. Shelby E. McDonald, shelby.e.mcdonald@colostate.edu) contingent upon appropriate data-use agreements and institutional approval.
