# Supplementary material for: Integrating animal cruelty exposure into person-centered models of childhood adversity: latent classes and associations with depression, anxiety, and stress
Source: Front Psychiatry. 2025 Nov 7;16:1701584. doi: 10.3389/fpsyt.2025.1701584 (PMC12634613; doi:10.3389/fpsyt.2025.1701584)
Supplement: Supplementary file 1 [file Supplementaryfile1.docx]

Supplementary Material

**Supplemental Table S1**

*Descriptive Statistics of ACEs Items (N = 1147)*

| **Item** | **Never** | **Rarely / Sometimes** | **Most of the time / Always** |
| --- | --- | --- | --- |
| **Did a parent, guardian, or other household member…** | | | |
| 1) Yell, scream, or swear at you, insult or humiliate you? | 321 (28.0%) | 629  (54.8%) | 197  (17.2%) |
| 2) Threaten to, or actually, abandon you or throw you out of the house? | 732  (63.8%) | 338  (29.5%) | 77  (6.7%) |
| 3) Spank, slap, kick, punch, or beat you up? | 411  (35.8%) | 603  (52.6%) | 133  (11.6%) |
| 4) Hit or cut you with an object, such as a stick (or cane), bottle, club, knife, whip, etc. | 805  (70.2%) | 265  (23.1%) | 77  (6.7%) |
| **Did someone ever…** | | | |
| 5) Touch or fondle you in a sexual way when you did not want them to? | 841  (73.3%) | 258  (22.5%) | 48  (4.2%) |
| 6) Make you touch their body in a sexual way when you did not want them to? | 859  (74.9%) | 247  (21.5%) | 41  (3.6%) |
| 7) Attempt oral, anal, or vaginal intercourse with you when you did not want them to? | 916  (79.9%) | 199  (17.3%) | 32  (2.8%) |
| 8) Actually have oral, anal, or vaginal intercourse with you when you did not want them to? | 948  (82.7%) | 161  (14.0%) | 38  (3.3%) |
| **Did you see or hear a parent or household member in your home…** | | | |
| 9) Being yelled at, screamed at, sworn at, insulted or humiliated? | 431 (37.6%) | 546  (47.6%) | 170  (14.8%) |
| 10) Being slapped, kicked, punched, or beaten up? | 702  (61.2%) | 343  (29.9%) | 102  (8.9%) |
| 11) Being hit or cut with an object, such as a stick (or cane), bottle, club, knife, whip, etc. | 867  (75.6%) | 234  (20.4%) | 46  (4.0%) |
| **Animal cruelty items** | | | |
| 12) Did you see or hear a parent, guardian, or other household member hurt a pet on purpose? | 860  (75.0%) | 243  (21.2%) | 44  (3.8%) |
| 13) Did you see or hear a sibling or another child in your household hurt a pet on purpose? | 898  (78.3%) | 205  (17.9%) | 44  (3.8%) |

**Supplemental Table S2**

*Fit indices for unconditional latent class models with 1-10 classes*

| ***k*** | **Par** | **LL** | **AIC** | **BIC** | **SABIC** | **VLMR-LRT *p-*value** | **LMR-LRT *p-*value** | **BLRT *p-*value** | **Entropy** | **Condition #** | **Smallest *n*** |
| --- | --- | --- | --- | --- | --- | --- | --- | --- | --- | --- | --- |
| 1 | 26 | -11322.89 | 22697.79 | 22828.96 | 22746.37 |  |  |  |  | 0.585E-02 | 100.0% |
| 2 | 53 | -9269.17 | 18644.34 | 18911.72 | 18743.38 | <.001 | <.001 | <.001 | .904 | 0.247E-02 | 33.3% |
| **3** | **80** | **-8627.27** | **17414.55** | **17818.14** | **17564.03** | **<.001** | **<.001** | **<.001** | **.887** | **0.881E-04** | **14.4%** |
| **4** | **107** | **-8288.03** | **16790.06** | **17329.86** | **16989.99** | **.004** | **.005** | **<.001** | **.892** | **0.180E-02** | **10.0%** |
| **5** | **134** | **-8072.96** | **16413.93** | **17089.95** | **16664.32** | **<.001** | **<.001** | **<.001** | **.879** | **0.141E-02** | **8.63%** |
| **6** | **161** | **-7910.24** | **16142.47** | **16954.70** | **16443.32** | **.001** | **.001** | **<.001** | **.896** | **0.588E-03** | **5.23%** |
| **7** | **188** | **-7814.55** | **16005.09** | **16953.54** | **16356.39** | **.283** | **.286** | **<.001** | **.878** | **0.253E-03** | **4.97%** |
| 8 | 215 | -7739.43 | 15908.85 | 16993.51 | 16310.60 | .173 | .174 | <.001 | .891 | 0.348E-03 | 4.88% |
| 9 | 242 | -7680.02 | 15844.03 | 17064.90 | 16296.23 | .621 | .622 | <.001 | .897 | 0.198E-08 | 3.23% |
| 10 | 269 | -7619.54 | 15777.08 | 17134.16 | 16279.73 | .046 | .046 | <.001 | .900 | 0.147E-08 | 2.27% |

*Note. N* = 1,147; *k* = number of classes, Par = number of parameters, LL = log likelihood, AIC = Akaike information criterion, BIC = Bayesian information criterion, SABIC = sample-size adjusted BIC, VLMR-LRT = Vuong-Lu-Mendell-Rubin likelihood ratio test, LMR-LRT = Lu-Mendell-Rubin likelihood ratio test, BLRT = bootstrapped likelihood ratio test. The bolded values indicate the candidate models explored. Model 7 was the best fitting ordinal-item model based on SABIC and LRTs. Additional candidate models with less classes were modeled to examine whether greater model parsimony would address class separation issues.

**Supplemental Figure S1**

*Item probability plot for the 7-class model (N = 1,147)*


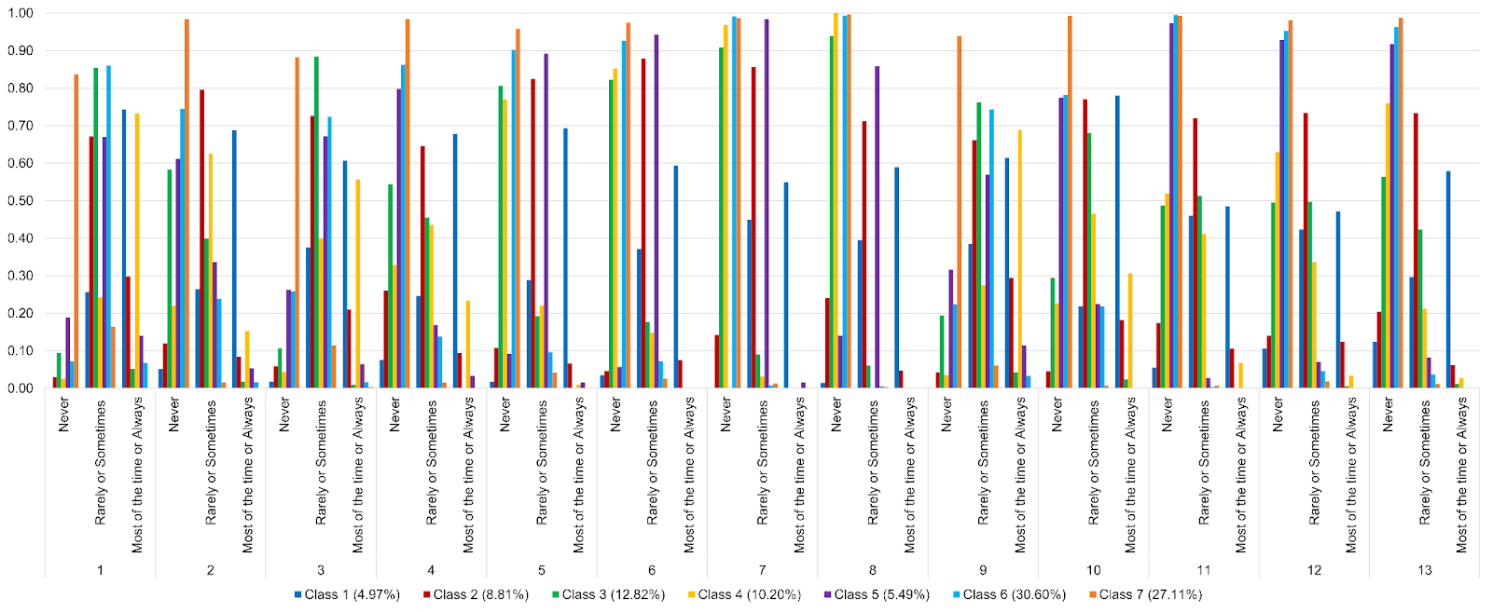


*Note.* The item numbers correspond with the items listed in Supplemental Table S1. This model was not selected due to most of the item probabilities falling between 0.30-0.70 in Class 1, which is considered “no greater than chance” for *rarely/sometimes* and *most of the time/always* across all indicators. Class 3 and Class 6 also shared similar item probabilities for items 1, 3, 5-8, and 9, which indicates poor class separation.

**Supplemental Figure S2**

*Item probability plot for the 6-class model (N = 1,147)*


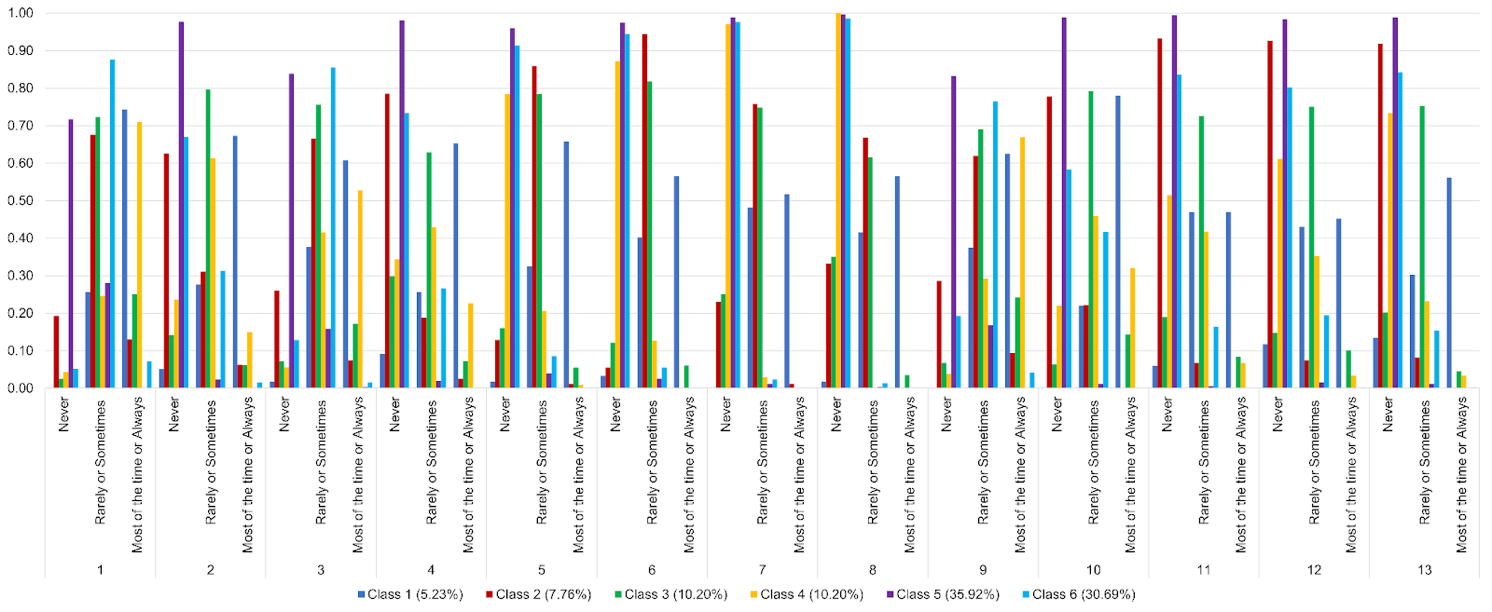


*Note.* The item numbers correspond with the items listed in Supplemental Table S1. This model was not selected due to most of the item probabilities falling between 0.30-0.70 in Class 1, which is considered “no greater than chance” for *rarely/sometimes* and *most of the time/always* across all indicators.

**Supplemental Figure S3**

*Item probability plot for the 5-class model (N = 1,147)*


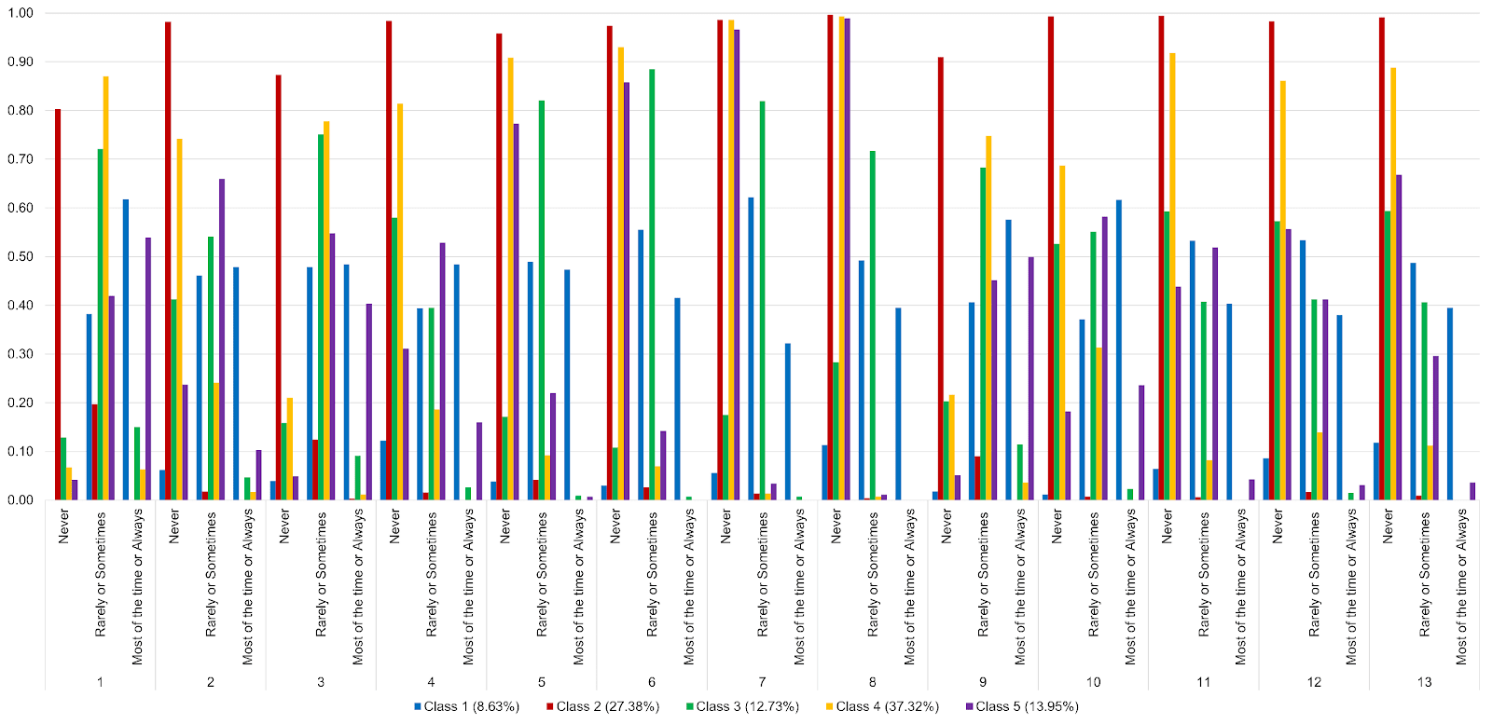


*Note.* The item numbers correspond with the items listed in Supplemental Table S1. This model was not selected due to most of the item probabilities falling between 0.30-0.70 in Class 5, which is considered “no greater than chance” for *rarely/sometimes* and *most of the time/always* across all indicators.

**Supplemental Figure S4**

*Item probability plot for the 4-class model (N = 1,147)*


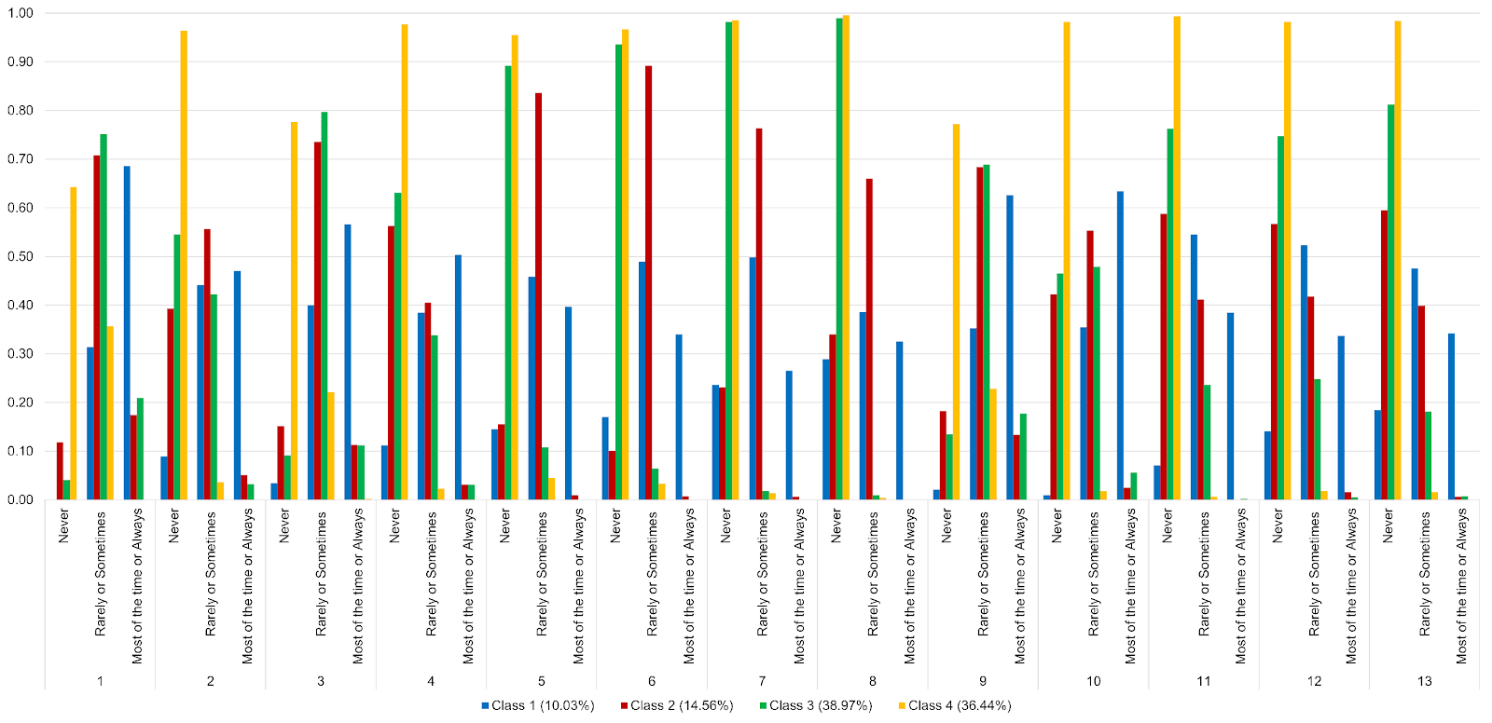


*Note.* The item numbers correspond with the items listed in Supplemental Table S1. This model was not selected due to most of the item probabilities falling between 0.30-0.70 in Class 1, which is considered “no greater than chance” for *rarely/sometimes* and *most of the time/always* across all indicators.

**Supplemental Figure S5**

*Item probability plot for the 3-class model (N = 1,147)*


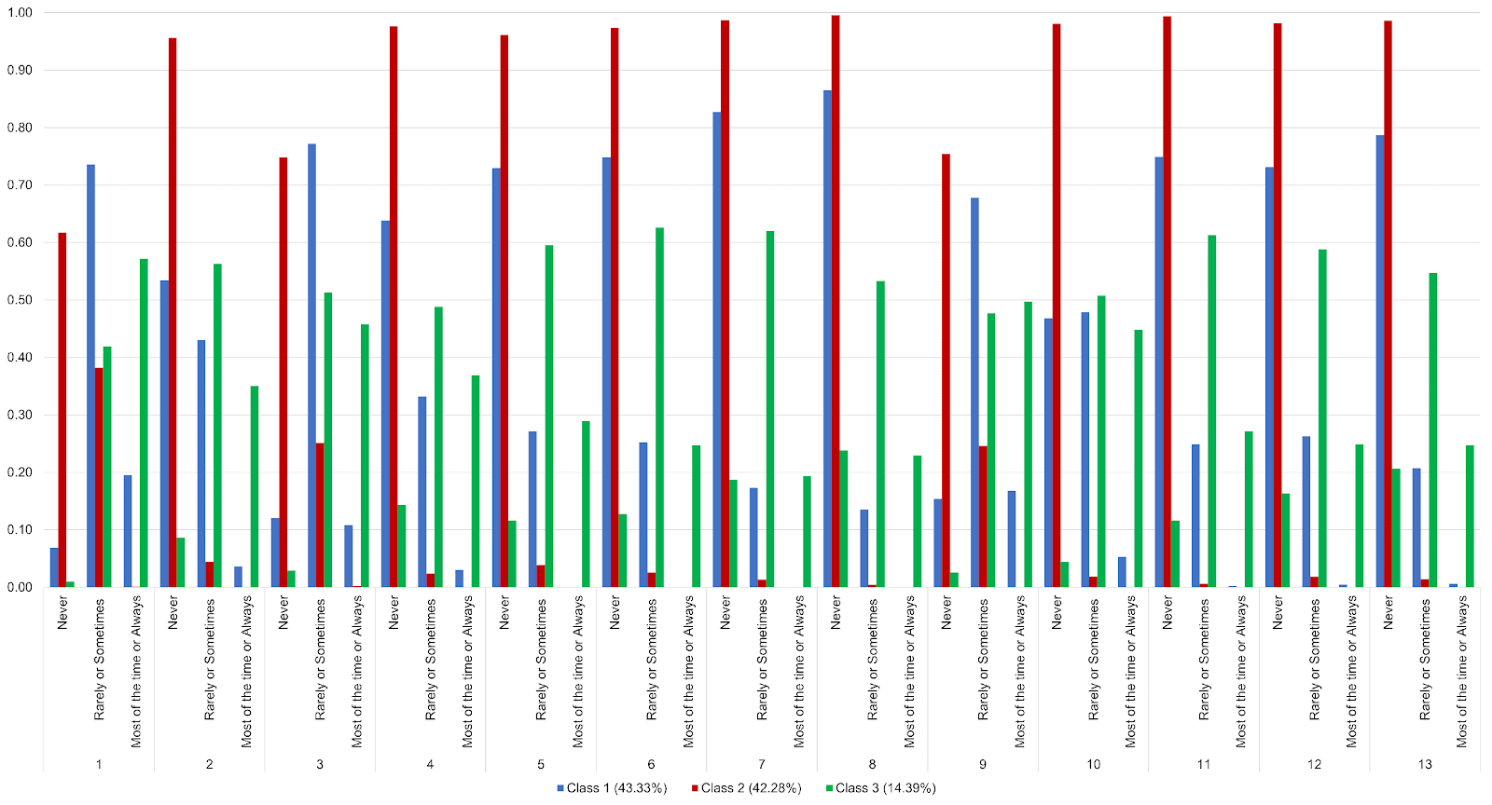


*Note.* The item numbers correspond with the items listed in Supplemental Table S1. This model was not selected due to most of the item probabilities falling between 0.30-0.70 in Class 3, which is considered “no greater than chance” for *rarely/sometimes* and *most of the time/always* across all indicators.
